# An oncoplastic technique to reduce the formation of lateral 'dog-ears' after mastectomy

**DOI:** 10.1186/1477-7800-4-29

**Published:** 2007-12-17

**Authors:** Haresh Devalia, Anushka Chaudhry, Richard M Rainsbury, Neda Minakaran, Dibyesh Banerjee

**Affiliations:** 1 St George's Hospital, Blackshaw Road, London, UK; 2 Royal Hampshire County Hospital, Winchester, Hants, UK

## Abstract

**Background:**

Lateral skin folds or 'dog-ears' are frequent following mastectomy, particularly in patients with large body habitus.

**Methods:**

We describe a method of modifying the mastectomy incision and suturing to eliminate these lateral 'dog-ears'.

**Conclusion:**

This surgical technique, as compared to others described in the literature, is simple, does not require additional incisions and is cosmetically acceptable to the patient.

## Background

Cosmetically sub-optimal lateral skin-folds or 'dog-ears' are frequent following mastectomy. Skin closure can be particularly challenging in patients with large body habitus. The resultant lateral 'dog-ears' tend to hang over the top of the brassiere which can be a nuisance. We illustrate an oncoplastic technique used to eliminate lateral 'dog-ears'.

## Methods

**Figure 1:**
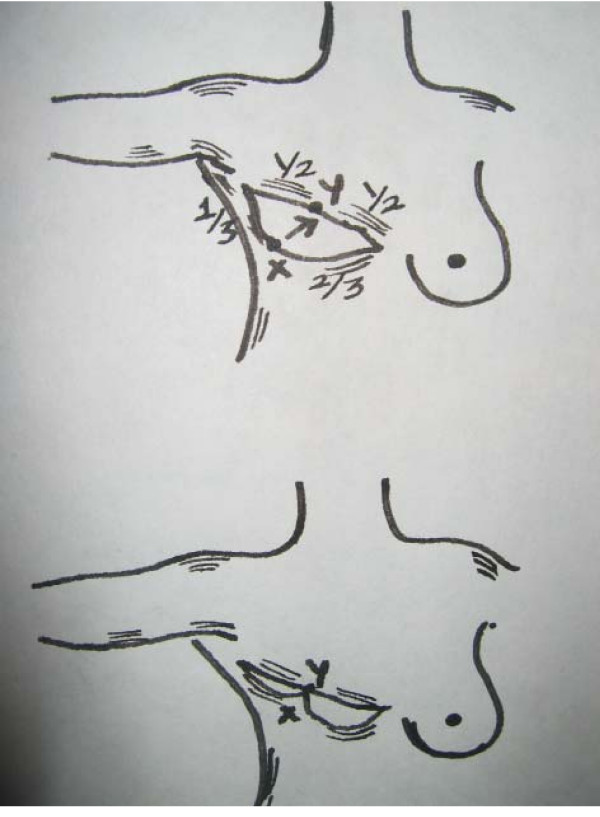
**Illustration of surgical technique to minimise 'dog-ears' in mastectomy**. The elliptical mastectomy incision is extended laterally and upwards towards the axilla. Following mastectomy point x is sutured to point y (please see text).

The pre-operative marking of the mastectomy incision normally involves drawing an ellipse of varying dimensions depending on the size of the breast. Our technique includes extending this ellipse laterally and upwards towards the axilla (Fig 1). This has the added advantage of flattening the lateral bulge that obese patients tend to develop. Following the mastectomy, a point (point x, Fig 1) approximately one third of the way from the lateral apex along the inferior aspect of the incision is sutured to a point more medially on the superior flap (point y, Fig 1). The lateral part of the incision can then be secured using dermal sutures, eliminating the 'dog-ear' and drawing the scar up into the axilla.

## Discussion

**Figure 2:**
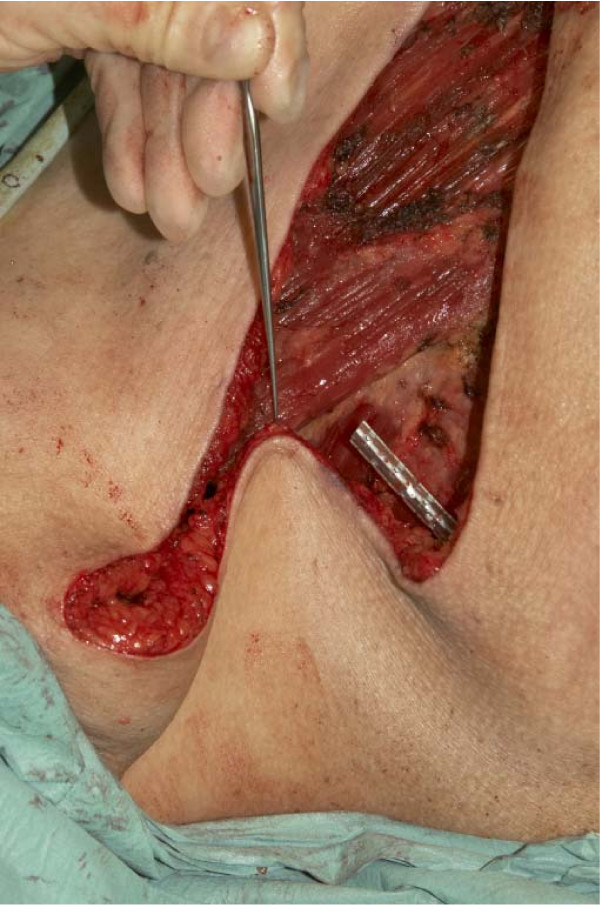
**Photograph of surgical technique to minimise 'dog-ears' in mastectomy**. Please see text.

**Figure 3:**
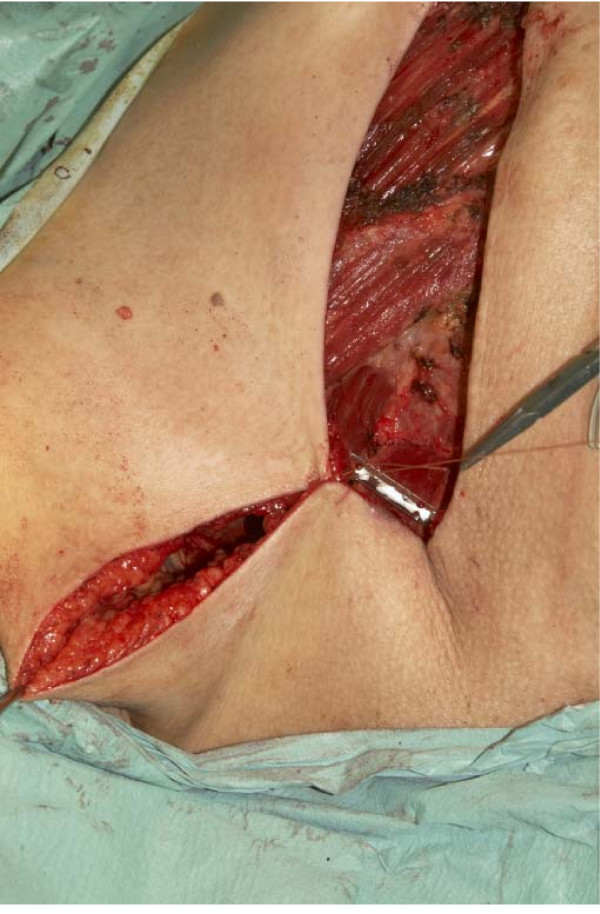
**Photograph of surgical technique to minimise 'dog-ears' in mastectomy**. Please see text.

Various surgical techniques e.g., fish-shaped incision, tear-drop incision have been described to eliminate 'dog-ear' deformity after mastectomy [1-3]. Our technique is simple and it does not involve additional incisions. The cosmetic outcome is more acceptable to patients, both aesthetically and for bra/prosthesis fitting. The clinical photographs (Fig 2) and (Fig 3) demonstrate this difference.

## Conclusion

The technique described can be used in oncoplastic surgery to improve cosmetic outcome.

## Competing interests

The author(s) declare that they have no competing interests.

## Authors' contributions

HD: Clinical studies, design, data acquisition, manuscript preparation, literature search.

AC: Data acquisition, manuscript preparation.

RMR: Concept, definition of intellectual content.

NM: Literature search, manuscript preparation.

DB: Clinical studies, manuscript editing, manuscript review.
